# Low awareness of proper use of cold and cough medication among Czech paediatricians: a questionnaire study

**DOI:** 10.1007/s00431-026-06937-z

**Published:** 2026-04-20

**Authors:** Ľuboš Bača, Eva Fürstová, Monika Pecková, Kateřina Kotíková, Jan David, Patrik Konopásek

**Affiliations:** 1 Department of Paediatrics, First Faculty of Medicine, Charles University, Thomayer University Hospital, Prague, Czech Republic; 2 Institute of Applied Mathematics and Information Technologies, Faculty of Science, Charles University, Prague, Czech Republic; 3 Toxicology Information Centre, First Faculty of Medicine, General University Hospitalin , Prague, Prague, Czech Republic; 4 Department of Children and Adolescents, Third Faculty of Medicine, Charles University and University Hospital Královské Vinohrady, Prague, Czech Republic

**Keywords:** Children, Common cold and cough medication, Inappropriate use

## Abstract

Cough and cold medications (CCMs) are widely used in paediatric practice despite limited evidence of efficacy and concerns regarding safety in young children. This study aimed to evaluate Czech paediatricians’ knowledge, attitudes, and recommendation practices related to CCMs and to identify factors associated with inappropriate use in children under 6 years of age. An anonymous questionnaire-based survey was distributed online to hospital paediatricians and general practitioners for children across the Czech Republic. The survey assessed demographic characteristics, patterns of CCMs use, knowledge of efficacy and safety, awareness of international recommendations, and attitudes toward potential restrictions and educational interventions. Associations were analysed using descriptive statistics, logistic regression, and Fisher’s exact test where appropriate. Of 266 respondents, 74.1% reported using CCMs in children under 6 years and 40.6% in children under 2 years. Recommendation practices were not associated with age or postgraduate training but varied significantly by region and medical school attended. Reliance on international literature was associated with the lowest use of CCMs, whereas reliance on local sources or older colleagues predicted higher use. Lack of awareness of the unproven efficacy of CCMs, as well as recommendations from the American Academy of Pediatrics and Health Canada, were the strongest predictors of inappropriate recommendations. While knowledge of the Medicines and Healthcare products Regulatory Agency recommendation also showed association with a good recommendation practice, the association was not significant in the multivariable model. *Conclusion: *Despite robust international guidance, CCMs remain commonly used in Czech paediatrics. Knowledge gaps, entrenched habits, and reliance on outdated information sources contribute to inappropriate use. Targeted educational initiatives and harmonised national guidelines are needed to improve practice.

**Table:** 

What is Known: ●* CCMs remain widely used in paediatrics despite limited efficacy and safety concerns in young children.* ● *International recommendations, including those from the AAP and Health Canada, advise against CCMs use in children under 6 years.*	
What is New: ●* In the Czech Republic, CCMs are used by 74% of paediatricians in children < 6 years and 41% in children < 2 years.* ●* CCMs recommendation practices vary by region and medical school, but not by age or training level. Higher inappropriate use is associated with reliance on local sources and older colleagues.*

**Supplementary Information:**

The online version contains supplementary material available at 10.1007/s00431-026-06937-z.

## Introduction

Cough and common cold are among the most frequent reasons for consultations in paediatric practice across Europe [1]. Despite their predominantly self-limiting nature, symptomatic treatment with cough and cold medications (CCMs)—including antitussives, expectorants, nasal decongestants, and antihistamines—remains widespread, largely due to their over-the-counter availability [1, 2].

However, the efficacy and safety of many CCMs in young children have been questioned, prompting several international regulatory bodies and professional societies to issue recommendations limiting their use, especially in children under 6 years of age [3–5]. Although CCMs are not recommended for young children by respected international authorities, their use remains high in some European countries. A regional primary care audit of CCMs in Spain showed that 85.1% of the prescribed medications were deemed unsuitable for the age group studied [6]. Moreover, data from Finland shows that the use of CCMs prescription is not affected by the official recommendation about age restriction [7].

We assume that in the Czech Republic (CZ) CCMs continue to be commonly recommended, yet there is a paucity of data regarding paediatricians’ knowledge and attitudes towards their appropriate use. Frequent use of CCMs in children poses not only the risk of unnecessary medication but also the possibility of incorrect dosing, which may lead to overdose or adverse events. In our recent study, we found a high prevalence of CCMs intoxications in children in the CZ, with the most cases occurring in children younger than 6 years of age [8]. Considering the documented risks associated with inappropriate administration, including adverse drug reactions and accidental intoxications, it is essential to assess current clinical practice and knowledge gaps among healthcare providers [8, 9]. As previously reported by Csonka et al., CCMs prescription rates can be significantly reduced by active interventions [10, 11]*.*

The primary aim of the present study is to evaluate the general knowledge of Czech paediatricians regarding the appropriate use of CCMs and to identify characteristics associated with inappropriate recommendations and use. The secondary aim is to initiate a wider discussion within the paediatric community on the topic and to enhance knowledge and awareness of the treatment’s risks and benefits.

## Materials and methods

We conducted a questionnaire-based study targeting both hospital paediatricians and general paediatricians to investigate their patterns of use and general knowledge of CCMs usage in children in CZ. The questionnaire was divided into multiple sections (Supplementary Material 1). First, basic demographics (questions 1–5), patterns of CCMs use, and primary sources of knowledge (questions 6–10) were investigated. After this section, participants were not allowed to return to previous questions. Second, general knowledge regarding the efficacy and safety profile of CCMs (questions 11–13), as well as awareness of international recommendations (questions 14–17), was assessed. Third, we evaluated whether the statements on efficacy and international recommendations were perceived as surprising (question 18) and assessed participants’ agreement with the proposed steps to improve general knowledge and establish clearer guidelines for CCMs use (questions 19–20).

The questionnaire was developed based on a review of the relevant literature and previously published surveys. Prior to distribution, the items were reviewed by experts in the field to ensure clarity and content relevance. The online questionnaire was created using the survey tool Survio. The link was subsequently distributed to paediatricians through multiple channels to reach as many respondents as possible. First, we contacted all major paediatric institutions and the chief paediatricians of numerous paediatric departments, asking them to share the link with their members and affiliated colleagues. The link was also shared in various paediatric groups on multiple social media platforms. Additionally, a person-to-person approach was employed, encouraging participants to forward the link to their fellow paediatricians. Prior to completing the questionnaire, participants were required to provide informed consent for the anonymous evaluation of their data and the use of the results for scientific purposes, such as publication in a scientific journal or presentation at a medical congress. The questionnaire link was accessible to participants for 31 days.

Once the data were collected, an initial screening of responses was performed. Based on the data distribution, groups of question responses were created, and further analyses conducted. Five medical schools with a low number of participants were merged into two regional groups, while the remaining four medical schools were analysed individually. To ensure anonymity, each was randomly assigned a single capital letter code (A–F). Evaluation of responses according to basic demographics included the following: age; primary employment setting; region; level of postgraduate paediatric training; and medical school (Table 1). Additional stratified analyses were performed using responses to the rest of the questions.

**Table 1:** Baseline characteristics of respondents

		***N***	%
Age	< 30	64	24.2%
	31–40	134	50.8%
	> 41	66	25.0%
Primary employment	University hospital	112	42.1%
	Other hospital	61	22.9%
	General practitioner for children and other employment	93	35.0%
Region	Prague, the capital city	93	35.0%
	Central Bohemia Region	42	15.8%
	Other Bohemia Regions	44	16.5%
	Moravian and Silesian Regions	87	32.7%
Medical school attended	A	30	11.3%
	B	31	11.7%
	C	56	21.1%
	D	65	24.4%
	E	50	18.8%
	F	34	12.8%
Current stage of your postgraduate paediatric training	Early stage of postgraduate training	56	21.1%
	Completed core medical training	47	17.7%
	Specialist physician	163	61.3%

Statistical analysis was conducted using RStudio. *P* values < 0.05 were considered statistically significant. Descriptive statistics were presented as absolute numbers and percentages. Logistic univariate and multivariate models were applied to assess the associations. In cases with zero events in any category, Fisher’s exact test was applied.

## Results

### Basic characteristics

Out of 620 participants who opened the link, 266 agreed with informed consent and finished the questionnaire, of which 2 refused to provide their age. The majority of respondents were from Prague and its surroundings, younger than 40 years, and board-certified. There was a similar distribution of university hospital and primary care paediatricians (Table 1).

### Associations between respondents’ demographics and CCMs use in children under 6 and 2 years of age

A total of 197 participants (74.1%) reported using CCMs in children younger than 6 years, and 108 participants (40.6%) reported using them in children younger than 2 years. Clinicians’ age, employment setting, and level of postgraduate training were not associated with CCMs use in these age groups. Significant associations were found for both groups with the region and medical school attended (Table 2). However, in the multivariable model, region was the only factor significantly associated with CCMs use in both age groups (Table 3).

**Table 2:** Logistic univariable models of CCMs use in children under 6 and 2 years of age and demographics. CCMs, cough and cold medications; OR, odds ratio

Association between CCMs use in children under 6 years of age and demographics				
Parameter	Options	Respondents using CCMs < 6 years	OR	***P***-value
Region	Prague, the capital city	63.4% (59/93)	1.00	0.0021
	Central Bohemia Region	73.8% (31/42)	1.62	
	Other Bohemia Region	70.5% (31/44)	1.37	
	Moravian and Silesian Regions	87.4% (76/87)	3.98	
Medical school attended	A	63.3% (19/30)	1.01	0.0073
	B	74.2% (23/31)	1.68	
	C	75.0% (42/56)	1.76	
	D	63.1% (41/65)	1.00	
	E	80.0% (40/50)	2.34	
	F	94.1% (32/34)	9.37	
Association between CCMs use in children under 2 years of age and demographics				
Parameter	Options	Respondents using CCMs < 2 years	OR	P-value
Region	Prague, the capital city	30.1% (28/93)	1.07	< 0.0001
	Central Bohemia Region	28.6% (12/42)	1.00	
	Other Bohemia Region	29.5% (13/44)	1.04	
	Moravian and Silesian Regions	63.2% (55/87)	4.30	
Medical school attended	A	20.0% (6/30)	1.00	0.0035
	B	35.5% (11/31)	2.20	
	C	50.0% (28/56)	4.00	
	D	33.8% (22/65)	2.05	
	E	38.0% (19/50)	2.45	
	F	64.7% (22/34)	7.33

**Table 3:** Multivariable logistic models of statistically significant parameters. AAP, American Academy of Pediatrics; CCMs, cough and cold medications; HC, Health Canada; MHRA, Medicines and Healthcare products Regulatory Agency; OR, odds ratio

Association between CCMs use in children under 6 years of age and demographics				
Parameter				***P***-value
Region				0.0021
Association between CCMs use in children under 2 years of age and demographics				
Parameter				***P***-value
Region				< 0.0001
Association between CCMs use in children < 6 years and knowledge				
Parameter	Proportion of respondents using CCMs in children < 6 years (with knowledge vs without knowledge)		OR	***P***-value
Knowledge of the unproven efficacy	68.3% (136/199) vs 91.0% (61/67)		< 3.8	0.0025
Knowledge of AAP recommendation	50.6% (42/83) vs 84.7% (155/183)		2.63	0.0046
Knowledge of HC recommendation	37.2% (16/43) vs 81.1% (181/223)		4.21	0.0004
Association between Knowledge of CCMs’ unproven efficacy and demographics				
Parameter	Option	Proportion of respondents with the knowledge	OR	***P***-value
Primary employment	University hospital	73.0% (82/112)	2.62	0.0069
	Other hospital	88.5% (54/61)	1.00	
	General practitioner for children and other employment	67.7% (63/93)	4.29	
Region	Prague, the capital city	80.0% (74/93)	1.05	0.0160
	Central Bohemia Region	85.7% (36/42)	1.00	
	Other Bohemia Region	77.3% (34/44)	2.03	
	Moravian and Silesian Regions	63.2% (55/87)	2.91	

### Association between CCMs use in children younger than 6 years and respondents’ knowledge

Respondents who considered CCMs safe and effective in general (question 7) were more likely to use CCMs in children younger than 6 years compared with those who considered it safe and effective only in children older than 6 years and compared with those who did not consider CCMs safe and effective in any age group (Supplementary Material 2).

**Fig. 1:**
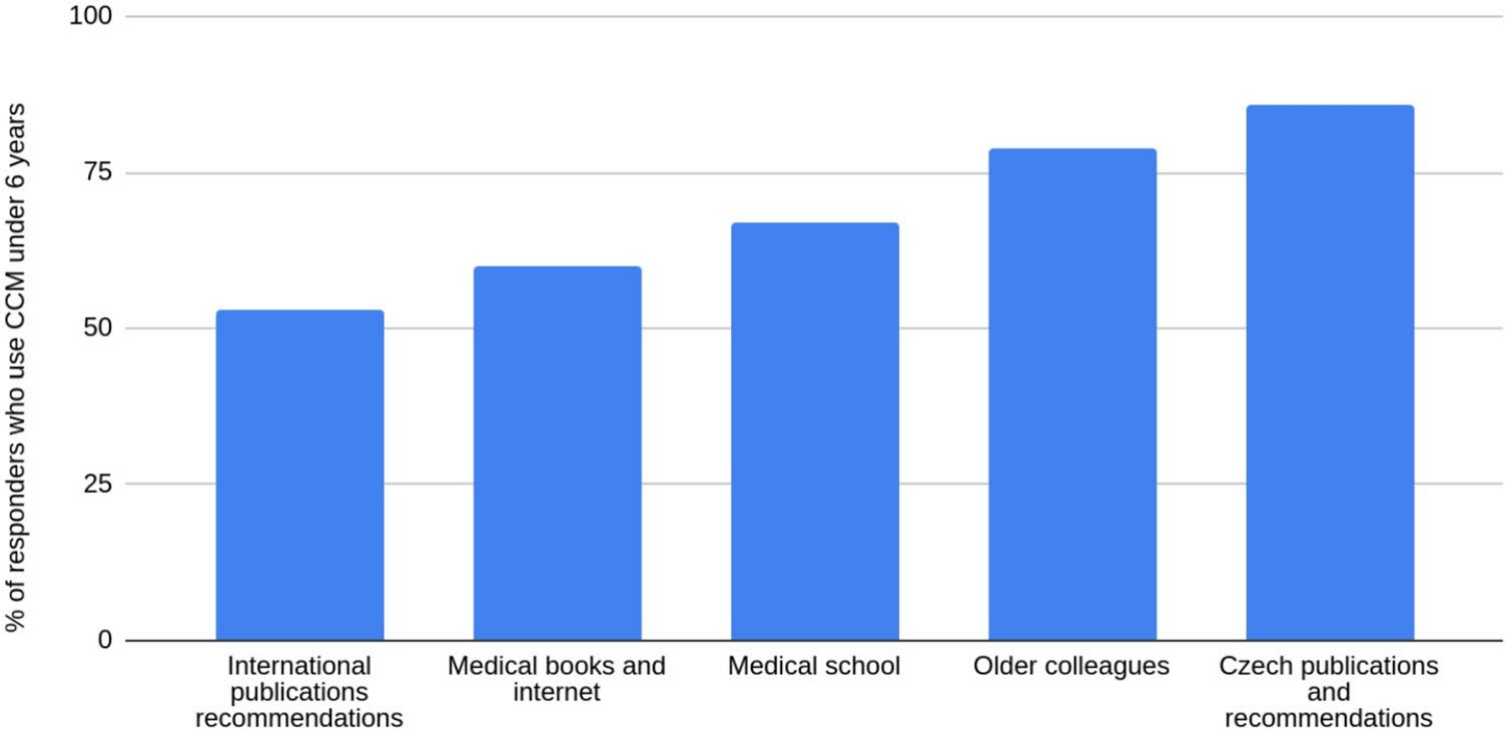
Association between the proportion of CCMs use in children under 6 years of age and the reported source of knowledge. International publications and recommendations (53%); medical books and internet (60%); medical school (67%); older colleagues (79%); Czech publications and recommendations (86%). P-value < 0.0001. CCMs, cough and cold medications

The primary source of knowledge (question 10) was significantly associated with CCMs use in children younger than 6 years. Respondents who relied primarily on international publications and guidelines reported the lowest use of CCMs in this age group, whereas CCMs use was highest among those who drew primarily on information from older colleagues or Czech publications and guidelines (Fig. 1).

Knowledge of the unproven efficacy of CCMs, the risk of adverse events in younger children, and international recommendations against CCMs use in children under 6 years of age (questions 11, 12, 14–17) were significantly associated with lower CCMs use in this age group (Supplementary Material 2). Despite these associations, a substantial proportion of respondents who reported having this knowledge still reported using CCMs in children younger than 6 years. Awareness of the Canadian recommendations was associated with less frequent CCMs use in this population (Supplementary Material 2). In the multivariable logistic model including questions 11–17, the strongest predictors of CCMs use in children under 6 years were lack of knowledge regarding CCMs efficacy, lack of awareness of the American Academy of Pediatrics (AAP) recommendations, and lack of awareness of Health Canada recommendations (Table 3). The remaining factors were not significant.

###  Associations between respondents’ demographics (questions 0–5) and their knowledge regarding CCMs (questions 11–17)

Knowledge of the unproven efficacy of CCMs (question 11) in children under 6 years of age was not associated with respondents’ age or level of postgraduate training, but it was associated with employment setting, region, and medical school attended (Supplementary Material 2). Clinicians from non-university hospitals, those from the Central Bohemia and Prague regions, and respondents from the A medical school demonstrated the best knowledge. In multivariable logistic models, only employment setting and region remained significant (Table 3).

Knowledge of the higher risk of adverse events in younger children (question 12) was not associated with employment setting, region, or medical school attended. However, we found an association with respondents’ age and level of postgraduate training. Older clinicians and those with a higher level of postgraduate training demonstrated better knowledge of the increased risk of CCMs in younger children (Supplementary Material 2). In multivariable logistic models, associations were observed with the level of postgraduate training and the medical school attended, while age was not significant in these models (Table 4).

**Table 4:** Multivariable logistic models of statistically significant parameters. AAP, American Academy of Pediatrics; CCMs, cough and cold medications; HC, Health Canada; MHRA, Medicines and Healthcare products Regulatory Agency; OR, odds ratio

Association between knowledge of the higher risk of CCMs-related adverse events in younger children and demographics				
Parameter	Option	Proportion of respondents with the knowledge	OR	***P***-value
Medical school attended	A	86.7% (26/30)	1.00	0.0488
	B	64.5% (20/31)	3.95	
	C	60.7% (34/56)	5.11	
	D	63.0% (41/65)	4.83	
	E	62.0% (31/50)	4.54	
	F	55.9% (19/34)	6.00	
Level of postgraduate training	Early stage of postgraduate training	50.0% (28/56)	2.71	0.0064
	Completed core medical training	60.0% (28/47)	1.83	
	Specialist physician	71.0% (115/163)	1.00	
Association between knowledge of severe CCMs-related adverse events including fatal cases and demographics				
Parameter	Option	Proportion of respondents with the knowledge	OR	***P***-value
Medical school attended	A	56.7% (17/30)	1.00	0.0013
	B	22.6% (7/31)	4.92	
	C	30.4% (17/56)	3.62	
	D	21.5% (14/65)	5.90	
	E	44.0% (22/50)	1.88	
	F	20.6% (7/34)	5.96	
Level of postgraduate training	Early stage of postgraduate training	16.1% (9/56)	3.53	0.0051
	Completed core medical training	36.0% (17/47)	1.26	
	Specialist physician	36.2% (58/163)	1.00	

Knowledge of severity of adverse effects resulting from CCMs use, including fatal cases (question 13), was associated with the level of postgraduate training (lower knowledge at lower levels) and the medical school attended (Supplementary Material 2). In the multivariable model, both parameters remained significant (Table 4).

Knowledge of the Food and Drug Administration (FDA) and Health Canada recommendations showed no association with any studied parameters. Knowledge of AAP recommendations was associated with the region only, with the highest awareness in Bohemia and Prague (Supplementary Material 2). The multivariable model confirmed this finding (*p* = 0.0291). Knowledge of the Medicines and Healthcare products Regulatory Agency (MHRA) recommendation was associated with region and medical school attended (Supplementary Material 2), but the multivariable model remained significant only for region (*p* = 0.0031). Knowledge of at least one recommendation showed no association in either model.

### Association with considering the recommendation on CCMs use as surprising

Only 39.8% of respondents considered the presented information surprising. Among all analysed parameters, being surprised by the recommendation on CCMs use was associated with many of the studied parameters (Supplementary Material 2). Based on the multivariable logistic regression model, significant associations remained only for use of CCMs in children younger than 6 years and younger than 2 years, lack of knowledge about the unproven efficiency, lack of knowledge of the risk of adverse events in younger children, lack of knowledge of the MHRA recommendation, and employment setting (Table 5).

**Table 5:** Multivariable logistic models of statistically significant parameters. AAP, American Academy of Pediatrics; CCMs, cough and cold medications; HC, Health Canada; MHRA, Medicines and Healthcare products Regulatory Agency; OR, odds ratio

Surprise at official positions on CCMs use and its associations				
Parameter	Proportion of respondents with surprise at official positions on CCMs use in selected groups		OR	***P***-value
Using CCMs under 2 years of age	63.9% (69/108)		2.37	0.0168
Using CCMs under 6 years of age	50.8% (100/197)		3.94	0.0001
Lack of knowledge of the unproven efficiency	77.6% (52/67)		6.24	< 0.0001
Lack of knowledge of the risk of adverse events in younger children	64.0% (61/95)		2.89	0.0017
Lack of knowledge of MHRA recommendation	50.8% (98/193)		4.98	0.0002
Parameter	Options	Proportion of respondents being surprised	OR	***P***-value
With primary employment	University hospital	33.0% (37/112)	1.00	0.0026
	Other hospital	42.6% (26/61)	3.92	
	General practitioner for children and other employment	46.2% (43/93)	2.69	
Disagreement with restrictions and its associations				
Parameter	Proportion of respondents with disagreement		OR	***P***-value
Respondents considering CCMs safe compared to those considering safe and effective just for children older than 6 years	46.3% (38/82) vs 12.1% (14/116)		5.13	< 0.0001
Respondents considering CCMs safe compared to those not considering CCMs safe and effective regardless of age	46.3% (38/82) vs 4.4% (3/68)		9.95	
Respondents using CCMs in children under 6 years compared to those not using CCMs in children under 6 years	27.0% (53/197) vs 2.9% (2/69)		4.35	0.0314

### Agreement with restrictions and information campaign (questions 19 and 20)

Among all respondents, 79.3% agreed that official restrictions should be implemented regarding CCMs use. No association was found between this agreement and basic demographic characteristics. A significant difference was observed between respondents who use CCMs in children under 6 and under 2 years of age and those who do not (Supplementary Material 2). Respondents who considered CCMs safe and effective in children under 6 years of age were most frequently opposed to any restrictions (Supplementary Material 2). Agreement with restrictions was not associated with the primary source of knowledge, awareness of the risk of severe adverse events, or awareness of foreign recommendations. However, it was associated with knowledge of the unproven efficacy of CCMs and awareness of a higher risk of harm from CCMs use in children under 6 years (Supplementary Material 2). Based on the multivariable logistic model, only CCMs used in children under 6 years and the belief that CCMs are safe and effective in all children were associated with disapproval of restrictions (Table 5).

Only 7% of respondents opposed large-scale information campaigns. Disapproval of the campaigns was associated with CCMs use in children under 6 years of age (respondents who disapproved were only within this category), CCMs use in children under 2 years of age, and lack of knowledge regarding both the unproven efficacy of CCMs and the AAP recommendation (Supplementary Material 2). In the multivariable logistic model, only CCMs use in children under 2 years of age remained significantly associated with campaign disapproval (OR 4.56, *p* = 0.0024).

## Discussion

This questionnaire study provides the first systematic assessment of Czech paediatricians’ knowledge, attitudes, and clinical practices regarding the use of CCMs in children. Despite well-established international recommendations [3–5] discouraging their use in young children under 6 years of age, CCMs remain a commonly administered medication. Nearly 75% of respondents reported using them in children under 6 years of age and over 40% in children under 2 years of age. Our findings highlight a substantial gap between current evidence and everyday paediatric practice in CZ.

Our findings are consistent with international reports from Europe and North America, where inappropriate use of CCMs has been documented despite unproven efficacy and ongoing safety concerns, suggesting that this issue is not limited to a single region [9, 12]. In a large Spanish study, Suárez-Castañón et al. show that 85% of prescribed CCMs were considered unsuitable for paediatric patients [6]. Moreover, a Finnish nationwide analysis demonstrated that publication of national guidelines did not reduce antitussive prescriptions [7].

In our study, recommendation and use practices were not associated with physicians’ age or level of postgraduate training, suggesting that the issue is not confined to a specific generation or training stage. However, significant regional and institutional differences were observed, implying a role of local educational traditions and institutional culture. We also found that the source of professional knowledge strongly affected CCMs recommendations and use. Respondents relying on international literature and evidence-based guidelines were less likely to use CCMs than those informed mainly by Czech sources or older colleagues. In our multivariable analysis, lack of familiarity with AAP and Health Canada recommendations was among the strongest predictors of inappropriate CCMs use. While knowledge of MHRA recommendations showed association in the univariable model, it was not significant in the multivariable model for inappropriate CCMs use. This suggests that possibly outdated national sources and the transmission of traditional practices among peers contribute to the persistence of inappropriate CCMs use. Alarmingly, we also observed that many paediatricians continued to recommend CCMs even when aware of their questionable efficacy and safety. This discrepancy suggests that knowledge alone may not suffice to change clinical practice/habits. Clinical habits and possible perceived parental demand may sustain inappropriate use despite awareness of risks.

Apart from the 2018 statement of the Czech Paediatric Society, issued at the request of the State Institute for Drug Control, which advises against the use of mucolytics in children under 2 years of age [13], no national recommendations are available in the CZ. In Europe, recent recommendations are available from the European Academy of Paediatrics within the Choosing Wisely initiative, which explicitly states: “Do not recommend, prescribe or use cough medicines in children.”, providing promising simple guidance to improve clinician’s practices [14]. In addition, specific national guidance is available from the UK MHRA, which has issued detailed recommendations on the use of cough and cold medicines in children.

The overall high acceptance of information campaigns among respondents indicates a readiness for targeted educational interventions. Not surprisingly, those who considered CCMs safe and effective and recommended them most frequently were also the most resistant to restrictions and campaigns. Experience from the USA and Canada demonstrates that regulatory and educational measures can reduce CCMs-related adverse events. Following the 2007–2008 FDA and industry actions, emergency department visits for CCMs-related adverse events declined significantly among children under 2 years [15]. Similarly, after Health Canada mandated “not for use under six years” labelling, over-the-counter CCMs sales and exposures in young children decreased [16]. In the Czech Republic, a similar labelling obligation has not yet been implemented. Despite already existing age warnings, inappropriate recommendation and use remain common, likely due to inconsistent education and deeply rooted local traditions. Focused dissemination of international safety alerts and evidence-based treatment guidelines could help align clinical practice with current recommendations. Future efforts should prioritise improving the education of medical students and young paediatricians. This can be supported by developing updated national guidelines and recommendations, ideally in collaboration with professional medical societies and national regulatory bodies. Evidence-based approaches to the use of CCMs should also be explicitly incorporated into undergraduate and postgraduate paediatrics curricula. Educational conferences should also focus particularly on regions where awareness of CCMs use is lower. As reported in Finland by Csonka et al. and Palmu et al. [11, 17], only targeted educational interventions successfully reduced CCMs prescribing rates. In addition, engaging pharmacists and other healthcare professionals in coordinated educational campaigns may further support safer, evidence-based indications and use of CCMs.

Several limitations should be acknowledged. First, the questionnaire was not formally pilot tested prior to administration, which may have introduced potential response bias. However, expert review and careful item construction were used to mitigate this risk. The voluntary nature of participation introduces possible selection bias; moreover, actual recommendations and use practices may differ from self-reported attitudes. Interpretation of our findings must take into account both the heterogeneity between pharmacological groups and the variability of individual medicines within each group, as agents may differ substantially in their efficacy and safety profiles. We also cannot exclude social desirability bias—respondents providing answers they consider professionally appropriate rather than reflecting their true knowledge or real clinical practice. We aimed for a nationwide survey; however, some Czech districts have a low response rate; therefore, we acknowledge the limited representativeness of the sample. A larger number of respondents would be needed to achieve better national representativeness, although the overall cohort is sufficient to generate statistically significant results.

## Conclusions

Despite clear international recommendations, CCMs remain widely used among Czech paediatricians, mainly due to limited awareness, entrenched habits, and outdated information sources. Education, guideline harmonisation, and targeted communication are essential, as labelling alone is unlikely to reduce inappropriate use in young children.

## Supplementary Information

Below is the link to the electronic supplementary material.

ESM 1 (DOCX 3.19 MB)

ESM 2 (DOCX 30.0 KB)

ESM 3 (XLSX 60.9 KB)

## Data Availability

Complete data are presented in the article and its supplementary materials.

## Abbreviations

AAP: American Academy of Pediatrics
CCMs: Cough and cold medications
CZ: Czech Republic
FDA: Food and Drug Administration
MRHA: Medicines and Healthcare products Regulatory Agency
