# Supplementary material for: Low awareness of proper use of cold and cough medication among Czech paediatricians: a questionnaire study
Source: Eur J Pediatr. 2026 Apr 20;185(5):280. doi: 10.1007/s00431-026-06937-z (PMC13095936; doi:10.1007/s00431-026-06937-z)
Supplement: Supplementary file 1 — (DOCX 3.19 MB) [file 431_2026_6937_MOESM1_ESM.docx]

**Supplementary Material 1 – Full questionaire**. First English translation (1.1), followed by original questionair in Czech 1.2.

- 1. **English**

**Informed consent**:

Dear doctor,

Thank you for taking the time to complete this brief anonymous questionnaire. By filling out and submitting the questionnaire, you agree that your responses may be processed by the project authors and presented in various forms, such as conference presentations, scientific publications, or similar formats.

**Demographic information**

1. How old are you?
2. <30 years
3. 31-40 years
4. 41-50 years
5. 51-60 years
6. >60 years

2.Your primary employment is at:

1. University hospital
2. Regional hospital
3. Other than regional or university hospital
4. General practitioner for children
5. Other

3. In which region do you work?

1. Prague, the capital city
2. Central Bohemia Region
3. South Bohemia Region
4. Plzeň Region
5. Karlovy Vary Region
6. Ústí nad Labem Region
7. Liberec Region
8. Hradec Králové Region
9. Pardubice Region
10. Vysočina Region
11. South Moravian Region
12. Zlín Region
13. Olomouc Region
14. Moravian-Silesian Region

4. Which medical school did you attend?

1. First Faculty of Medicine, Charles University
2. Second Faculty of Medicine, Charles University
3. Third Faculty of Medicine, Charles University
4. Faculty of Medicine in Hradec Králové, Charles University
5. Faculty of Medicine in Pilsen, Charles University
6. Faculty of Medicine, University of Ostrava
7. Faculty of Medicine, Masaryk University
8. Faculty of Medicine and Dentistry, Palacký University Olomouc
9. Military Faculty of Medicine, University of Defence

5. What is the current stage of your postgraduate paediatric training?

1. Early stage of postgraduate training
2. Completed core medical training
3. Specialist physician

**Basic Questions**

6. For which paediatric age groups do you prescribe or recommend the use of antitussives, antihistamines, expectorants, mucolytics, and/or decongestants in the management of cough and the common cold?

1. In all age groups
2. Only in children older than 3 months
3. Only in children older than 2 years
4. Only in children older than 6 years
5. I never recommend or use this therapy

7. Do you consider antitussives, antihistamines, expectorants, mucolytics, and decongestants to be effective and safe treatments for cough and the common cold in children, including those under 6 years of age?

1. Yes.
2. Yes, but only in older children
3. No, I do not consider these medications to be effective and safe in the treatment of cough and the common cold, regardless of age.

8. Do you recommend the concurrent administration of mucolytics and expectorants with antitussives?

1. Yes.
2. No.

9. Do you inform parents about the risks associated with the use of antitussives, antihistamines, expectorants, mucolytics, and decongestants, particularly in younger children?

1. Yes.
2. No.

10. What is your primary source of information regarding the appropriate use of antitussives, antihistamines, expectorants, mucolytics, and decongestants in the treatment of cough and the common cold in children?

1. Knowledge acquired during medical school.
2. Textbook of paediatrics
3. Recommendations from a Czech professional society or an article in a Czech medical journal
4. Recommendations from international/foreign professional societies or foreign medical literature
5. Clinical approaches learned from a more experienced colleague
6. Commonly used internet search engine
7. This is common knowledge; I was aware of it before studying medicine.

**Section two, with no option to go back to previous questions.**

11. Are you aware that, according to randomized studies, antihistamines, antitussives, expectorants, mucolytics, and decongestants have not been shown to be effective in the treatment of cough and the common cold in young children, or that their effect has not been proven to be superior to placebo?

1. Yes.
2. No.

12. Are you aware that, in young children, the immaturity of detoxification mechanisms increases the risk of harmful effects associated with antihistamines, antitussives, expectorants, mucolytics, and decongestants?

1. Yes.
2. No.

13. Are you aware that, while antihistamines, antitussives, expectorants, mucolytics, and decongestants have not been proven effective in the treatment of cough and the common cold in young children, there have been reported cases of serious intoxications, including rare cases of death?

1. Yes.
2. No.

14. Are you aware that in 2008, the U.S. Food and Drug Administration (FDA) issued a recommendation that over-the-counter cough and cold medications should not be administered to children under 2 years of age, based on insufficient evidence of efficacy and documented risks of serious adverse reactions, and that this initiated a broader discussion about restricting their use in children under 12 years of age?

1. Yes.
2. No.

15. Are you aware that the American Academy of Pediatrics (AAP) does not recommend the use of antitussives, antihistamines, expectorants, or mucolytics in children under 6 years of age, and decongestants in children under 12 years of age?

1. Yes.
2. No.

16. Are you aware that since 2009, the UK Medicines and Healthcare products Regulatory Agency (MHRA) has not recommended the use of most of the above-mentioned medications in children under 6 years of age?

1. Yes.
2. No.

17. Are you aware that since 2009, Health Canada — the federal institution responsible for medical care quality — has not recommended the use of the above-mentioned medications in children under 6 years of age?

1. Yes.
2. No.

18. Do you find the above-mentioned positions of major professional societies and regulatory authorities surprising?

1. Yes.
2. No.

19. Do you agree that the Czech Republic should implement similar restrictions on the use of these medications for cough and the common cold?

1. Yes.
2. No.

20. Do you agree that a large-scale information campaign should be launched in the Czech Republic to educate both healthcare professionals and the general public about the risks and lack of proven efficacy of the above-mentioned medications in children under 6 years of age?

1. Yes.
2. No.

Dear Doctor,

Thank you very much for completing this questionnaire. Discussions regarding the use of medications for cough and the common cold have been ongoing for decades. The aforementioned professional institutions have begun addressing the issue by restricting the sale of these medications for young children.

In the Czech Republic, there is a high incidence of accidental intoxications related to these medications, with the primary affected group being children under 6 years of age. Poisonings from cough medications in the paediatric population account for 15.6% of all accidental drug intoxications across all age groups.

More detailed data can be found in our recent publication:
Konopásek P, Kotíková K, Pecková M, David J. High prevalence of cough and common cold medication intoxications in the Czech paediatric population. Klin Padiatr. 2025 Feb 7. English. doi: 10.1055/a-2518-5571.

Despite these facts, awareness of this issue remains low in the Czech Republic among both healthcare professionals and the general public.

**1.2. Czech language original**

**Vstupní poučení** (informovaný souhlas):

Vážená paní doktorko, vážený pane doktore,

děkujeme, že nám věnujete Váš čas vyplněním tohoto krátkého anonymního dotazníku. Vyplněním dotazníku a jeho odesláním souhlasíte, že odpovědi budou zpracovány autory projektu a mohou být prezentovány v různé podobě, např. formou kongresového sdělení, odborného článku apod.

**Demografické údaje**

1. Jaký je Váš věk?

1. <30 let
2. 31-40 let
3. 41-50 let
4. 51-60 let
5. >60 let

2. Váš hlavní pracovní úvazek máte ve:

1. Fakultní nemocnici
2. Krajské nemocnici
3. Jiné než krajské nebo fakultní nemocnici
4. Jako praktický lékař pro děti a dorost
5. Jiné

3. V jakém kraji pracujete?

1. Hlavní město Praha
2. Středočeský kraj
3. Jihočeský kraj
4. Plzeňský kraj
5. Karlovarský kraj
6. Ústecký kraj
7. Liberecký kraj
8. Královéhradecký kraj
9. Pardubický kraj
10. Kraj Vysočina
11. Jihomoravský kraj
12. Zlínský kraj
13. Olomoucký kraj
14. Moravskoslezský kraj

4. Na jaké lékařské fakultě jste studoval/a?

1. 1. lékařská fakulta Univerzity Karlovy
2. 2. lékařská fakulta Univerzity Karlovy
3. 3. lékařská fakulta Univerzity Karlovy
4. Lékařská fakulta Univerzity Karlovy v Hradci Králové
5. Lékařská fakulta Univerzity Karlovy v Plzni
6. Lékařská fakulta Ostravské univerzity v Ostravě
7. Lékařská fakulta Masarykovy univerzity
8. Lékařská fakulta Univerzity Palackého
9. Fakulta vojenského zdravotnictví Univerzity obrany

5. V jakém stádiu je Vaše postgraduální vzdělání v pediatrii?

1. Lékař před kmenem
2. Lékař s absolvovaným kmenem
3. Atestovaný lékař

**Základní otázky**

6. Pro jakou věkovou kategorii používáte / doporučujete na léčbu kašle a běžného nachlazení antitusika, antihistaminika, expektorancia, mukolytika a/nebo dekongescenční léky?

1. Ve všech věkových kategoriích.
2. Pouze u dětí nad 3 měsíce.
3. Pouze u dětí nad 2 roky.
4. Pouze u dětí nad 6 let.
5. Tuto terapii nikdy nedoporučuji ani nepoužívám.

7. Považujete antitusika, antihistaminika, expektorancia, mukolytika a dekongescenční léky za efektivní a bezpečná léčiva v terapii kašle a běžného nachlazení u dětí, a to včetně u dětí pod 6 let věku?

1. Ano.
2. Ano, ale pouze u starších dětí.
3. Ne, tyto léky nepovažuji za efektivní a bezpečné v terapii kašle a běžného nachlazení bez ohledu na věk.

8. Doporučujete současné podávání mukolytik a expektorancií s antitusiky?

1. Ano.
2. Ne.

9. Upozorňujete rodiče na rizika spojená s užíváním antitusik, antihistaminik, expektorancii, mukolytik a dekongescenčních léků, a to především u menších dětí?

1. Ano.
2. Ne.

10. Jaký je Váš primární zdroj informací týkající se správného použití antitusik, antihistaminik, expektorancií, mukolytik a dekongescenčních léků v terapii kašle a běžného nachlazení u dětí?

1. Znalosti ze studia medicíny.
2. Učebnice pediatrie.
3. Doporučení české odborné společnosti nebo článek v českém časopise.
4. Doporučení mezinárodní nebo zahraniční odborné společnosti nebo zahraniční odborná literatura.
5. Postupy naučené od zkušenějšího kolegy.
6. Běžně používaný internetový vyhledávač.
7. Jedná se o všeobecně známé informace, znalost jsem měl/a před studiem medicíny.

**Část druhá, bez možnosti návratu k předchozím otázkám.**

11. Věděl/a jste, že dle randomizovaných studií nemají u menších dětí antihistaminika, antitusika, expektorancia, mukolytika a dekongescenční léky prokázaný efekt v terapii kašle a běžného nachlazení resp. nebyl prokázán lepší účinek než placebo?

1. Ano.
2. Ne.

12. Věděl/a jste, že u malých dětí je v důsledku nezralosti detoxifikačních mechanismů vyšší riziko škodlivých účinků antihistaminik, antitusik, expektorancií, mukolytik a dekongescenčních léků?

1. Ano.
2. Ne.

13. Věděl/a jste, že zatímco antihistaminika, antitusika, expektorancia, mukolytika a dekongescenční léky nemají prokázány efekt v terapii kašle a běžného nachlazení u malých dětí, existují případy závažných intoxikací, a to včetně vzácných případů úmrtí?

1. Ano.
2. Ne.

14. Věděl/a jste, že v roce 2008 vydal Americký ústav pro kontrolu léčiv (FDA) doporučení, aby se běžně prodejné léky na kašel a nachlazení nepodávaly dětem pod 2 roky, a to na základě nepřesvědčivých datech o účinku a prokázanému riziku závažných nežádoucích reakcí, přičemž zahájilo debatu o zákazu těchto léků u dětí pod 12 let věku?

1. Ano.
2. Ne.

15. Věděl/a jste, že Americká pediatrická asociace (AAP) nedoporučuje podávání antitusik, antihistaminik, expektorancií, mukolytik u dětí do 6 let a dekongescenčních léků u dětí do 12 let?

1. Ano.
2. Ne.

16. Věděl/a jste, že britský ústav pro kontrolu léčiv (The Medicine and Healthcare Products Regulatory Agency) od roku 2009 nedoporučuje užívání většiny výše zmíněných léků u dětí do 6 let?

1. Ano.
2. Ne.

17. Věděl/a jste, že kanadská federální instituce zajišťující kvalitu lékařské péče (Health Canada) nedoporučuje od roku 2009 užívání výše zmíněných léků u dětí pod 6 let?

1. Ano.
2. Ne.

18. Jsou pro Vás výše zmíněná stanoviska významných odborných společností a institucí překvapivá?

1. Ano.
2. Ne.

19. Souhlasíte, že by v České republice měly být zavedeny obdobné restrikce stran užívání výše zmíněných léků na kašel a běžné nachlazení?

1. Ano.
2. Ne.

20. Souhlasíte, aby se v České republice zahájila masivní informační kampaň s cílem edukovat lékaře a laickou veřejnost o rizicích a neprokázaném efektu výše zmíněných léků u dětí do 6 let?

1. Ano.
2. Ne.

Vážená paní doktorko, vážený pane doktore, moc děkujeme za vyplnění tohoto dotazníku. Desítky let se již vedou diskuze o užívání léků na kašel a běžného nachlazení. Zmíněné odborné instituce situaci začaly řešit omezením prodeje těchto léků u malých dětí. V České republice je vysoká incidence náhodných intoxikací výše uvedenými léky, přičemž hlavní skupinu těchto intoxikací tvoří děti do 6 let. Intoxikace léky na kašel v pediatrické populaci pak tvoří 15.6 % všech náhodných medikamentózních intoxikací ze všech věkových kategorií. Podrobnější data lze nález v naší čerstvé publikaci (Konopásek P, Kotíková K, Pecková M, David J. High prevalence of cough and common cold medication intoxications in the Czech paediatric population. Klin Padiatr. 2025 Feb 7. English. doi: 10.1055/a-2518-5571). I přes výše uvedená fakta v České republice přetrvává nízké povědomí ohledně této problematiky jak u odborné, tak u laické veřejnosti.
