# Supplementary material for: Low awareness of proper use of cold and cough medication among Czech paediatricians: a questionnaire study
Source: Eur J Pediatr. 2026 Apr 20;185(5):280. doi: 10.1007/s00431-026-06937-z (PMC13095936; doi:10.1007/s00431-026-06937-z)
Supplement: Supplementary file 2 — (DOCX 30.0 KB) [file 431_2026_6937_MOESM2_ESM.docx]

**Supplementary Material 2 – Logistic univariable models of statistically significant parameters. The category associated with the lowest risk was used as the reference group and was assigned OR of 1.** AAP – American Academy of Pediatrics; CCMs – cough and cold medications; FDA – Food and Drug Administration; HC – Health Canada; MHRA – Medicines and Healthcare products Regulatory Agency; OR – odds ratio.

| **Association between CCMs use in children <6 years and perceptions of their safety and effectiveness** | | | | | |
| --- | --- | --- | --- | --- | --- |
| **Parameter** | | **Respondents using CCMs < 6 years** | **OR** | **P - value** |  |
| Respondents considering CCMs safe and effective in general compared to those considering CCMs safe and effective only for children older than 6 years | | 99.0% vs 77.0% | 25 | < 0.0001 |  |
| Respondents considering CCMs safe and effective in general compared to those not considering CCMs safe and effective regardless of age | | 99.0% vs 40.0% | 123 | < 0.0001 |  |
| **Association between CCMs use in children <6 years and knowledge** | | | | | |
| **Parameter** | | **Respondents using CCMs < 6 years (knowledge absent vs present)** | **OR** | **P - value** |  |
| Knowledge of the unproven efficacy | | 91.0% vs 68.3% | 4.71 | < 0.0001 |  |
| Knowledge of the risk of intoxication in younger children | | 87.4% vs 66.7% | 3.46 | 0.0001 |  |
| Knowledge of FDA recommendation | | 79.4% vs 66.0% | 1.98 | 0.0158 |  |
| Knowledge of AAP recommendation | | 84.7% vs 50.6% | 5.40 | < 0.0001 |  |
| Knowledge of MHRA recommendation | | 83.4% vs 49.3% | 5.17 | < 0.0001 |  |
| Knowledge of HC recommendation | | 81.1% vs 37.2% | 7.27 | < 0.0001 |  |
| Knowledge of at least one recommendation | | 85.7% vs 63.6% | 3.44 | < 0.0001 |  |
| **Association between the knowledge of CCMs’s unproven efficacy and demographics** | | | | | |
| **Parameter** | **Option** | | **OR** | **P - value** |  |
| Primary employment | University hospital | | 2.88 | 0.0080 |  |
|  | Other hospital | | 1.00 |  |  |
|  | General practitioner for children and other employment | | 3.67 |  |  |
| Region | Prague, the capital city | | 1.54 | 0.0185 |  |
|  | Central Bohemia Region | | 1.00 |  |  |
|  | Other Bohemia Region | | 1.76 |  |  |
|  | Moravian Region | | 3.49 |  |  |
| Medical school attended | A | | 1.0 | 0.0297 |  |
|  | B | | 2.16 |  |  |
|  | C | | 3.29 |  |  |
|  | D | | 2.25 |  |  |
|  | E | | 3.86 |  |  |
|  | F | | 7.11 |  |  |
| **Association between knowledge of the higher risk of CCMs related intoxications in younger children and demographics** | | | | | |
| **Parameter** | **Options** | | **OR** | **P - value** |  |
| Age | < 30 | | 2.59 | 0.0378 |  |
|  | 31 – 40 | | 1.80 |  |  |
|  | > 41 | | 1.00 |  |  |
| Level of postgraduate training | Early stage of postgraduate training | | 2.40 | 0.0175 |  |
|  | Completed core medical training | | 1.63 |  |  |
|  | Specialist physician | | 1.00 |  |  |
| **Association between knowledge of severe intoxications resulting from CCMs use and demographics** | | | | | |
| **Parameter** | **Options** | | **OR** | **P - value** |  |
| Medical school attended | A | | 1.00 | 0.0030 |  |
|  | B | | 4.48 |  |  |
|  | C | | 3.00 |  |  |
|  | D | | 4.76 |  |  |
|  | E | | 1.66 |  |  |
|  | F | | 5.04 |  |  |
| Level of postgraduate training | Early stage of postgraduate training | | 2.96 | 0.0131 |  |
|  | Completed core medical training | | 1.00 |  |  |
|  | Specialist physician | | 1.03 |  |  |
| **Association between knowledge of AAP reccomendation and demographics** | | | | | |
| **Parameter** | **Options** | | **OR** | **P - value** |  |
| Region | Prague, the capital city | | 1.26 | 0.0291 |  |
|  | Central Bohemia Region | | 1.25 |  |  |
|  | Other Bohemia Region | | 1.00 |  |  |
|  | Moravian Region | | 2.85 |  |  |
| **Association between knowledge of MHRA reccomendation and demographics** | | | | | |
| **Parameter** | **Options** | | **OR** | **P - value** |  |
| Region | Prague, the capital city | | 1.36 | 0.0031 |  |
|  | Central Bohemia Region | | 1.00 |  |  |
|  | Other Bohemia Region | | 1.08 |  |  |
|  | Moravian Region | | 3.85 |  |  |
| Medical school attended | A | | 1.00 | 0.0267 |  |
|  | B | | 1.84 |  |  |
|  | C | | 3.58 |  |  |
|  | D | | 2.28 |  |  |
|  | E | | 1.86 |  |  |
|  | F | | 6.56 |  |  |
| **Disagreement with restrictions and its associations** | | | | | |
| **Parameter** | | **Respondents who disagree (yes vs. no)** | **OR** | **P - value** |  |
| Using CCMs under 2 years of age | | 33.3% vs 12.9% | 3.66 | < 0.0001 |  |
| Using CCMs under 6 years of age | | 26.9% vs 2.9% | 12.33 | < 0.0001 |  |
| Respondents considering CCMs safe compared to those not considering CCMs safe and effective regardless of age | | 46.3% vs 4.4% | 6.29 | < 0.0001 |  |
| Respondents considering CCMs safe only for children older than 6 years compared to those not considering CCMs safe and effective regardless of age | | 12.1% vs 4.4% | 18.71 | < 0.0001 |  |
| Lack of knowledge of the unproven efficacy | | 37.3 vs 15.1% | 3.35 | 0.0002 |  |
| Lack of knowledge of the higher risk of harm from CCMs in younger children | | 27.4% vs 17.0% | 1.85 | 0.0475 |  |
| **Disapproval with large-scale information campaigns and its associations** | | | | | |
| **Parameter** | | **Respondents who disagree (yes vs. no)** | **OR** | **P - value** |  |
| Using CCMs under 2 years of age | | 13.0% vs 3.2% | 4.56 | 0.0024 |  |
| Using CCMs under 6 years of age (Fisher test) | | 9.6% vs 0.0% | - | 0.0048 |  |
| Lack of knowledge of the unproven efficiency | | 13.4% vs 5.0% | 2.93 | 0.0298 |  |
| Lack of knowledge of the AAP recommendation | | 9.0% vs 2.4% | 4.15 | 0.0272 |  |
| **Surprise at official positions on CCMs use and its associations (multiple options questions)** | | | | | |
| **Parameter** | **Options** | **Surprised respondents** | **OR** | **P - value** |  |
| Medical school attended | A | 23% | 1.00 | 0.0327 |  |
|  | B | 45% | 2.71 |  |  |
|  | C | 41% | 2.29 |  |  |
|  | D | 29% | 1.36 |  |  |
|  | E | 48% | 3.03 |  |  |
|  | F | 56% | 4.16 |  |  |
| Respondents primary source of knowledge | International publications recommendations | 15.6% | 1.00 | < 0.0001 |  |
|  | Medical books and internet | 60.0% | 8.10 |  |  |
|  | Medical school | 38.9% | 3.44 |  |  |
|  | Older colleagues | 58.0% | 7.48 |  |  |
|  | Czech publications and recommendations | 42.0% | 3.90 |  |  |
| **Surprise at official positions on CCMs use and its associations (2 options questions)** | | | | |  |
| **Parameter** | | **Surprised respondents (yes vs. no)** | **OR** | **P - value** |  |
| Using CCMs under 6 years of age | | 50.8% vs 8.7% | 10.82 | < 0.0001 |  |
| Using CCMs under 2 years of age | | 63.9% vs 23.4% | 5.79 | < 0.0001 |  |
| Respondents considering CCMs safe compared to those not considering CCMs safe and effective regardless of age | | 63.4% vs 25.0% | 5.20 | < 0.0001 |  |
| Respondents considering CCMs safe only for children older than 6 years compared to those not considering CCMs safe and effective regardless of age | | 31.9% vs 25.0% | 1.41 | < 0.0001 |  |
| Lack of knowledge of the unproven efficacy | | 77.6% | 9.31 | < 0.0001 |  |
| Lack of knowledge of the higher risk of harm from CCMs | | 64.2% | 5.02 | < 0.0001 |  |
| Lack of knowledge of the risk of intoxication in younger children | | 46.7% | 2.63 | 0.0006 |  |
| Lack of knowledge of FDA recommendation | | 51.9% | 3.89 | < 0.0001 |  |
| Lack of knowledge of AAP recommendation | | 50.8% | 5.56 | < 0.0001 |  |
| Lack of knowledge of MHRA recommendation | | 50.8% | 8.38 | < 0.0001 |  |
| Lack of knowledge of HC recommendation | | 44.8% | 5.01 | < 0.0001 |  |
